# Black tendon—identifying a rare autosomal recessive disorder: Intraoperative diagnosis of alkaptonuria: A case report

**DOI:** 10.1177/2050313X241283597

**Published:** 2024-10-11

**Authors:** Parag Pushpan, Sai Harsha Sreepada, Kiran K V Acharya, Jonathan Abraham

**Affiliations:** Department of Orthopaedics, Kasturba Medical College, Manipal, Karnataka, India

**Keywords:** Calcaneal avulsion, alkaptonuria, ochronosis, tendon degeneration, spontaneous avulsion, Achilles

## Abstract

Tendon injuries in the lower extremities are common in physically active individuals, although spontaneous Achilles tendon ruptures are linked to oral corticosteroid or fluoroquinolone use. Such ruptures are typically due to degenerative changes within the tendon and frequently occur during sudden dorsiflexion of a plantar-flexed foot. Bilateral spontaneous ruptures are especially prevalent in patients undergoing long-term corticosteroid therapy. Here, we present a rare case of bilateral spontaneous calcaneal avulsion in an otherwise healthy woman, ultimately diagnosed with alkaptonuria. This case underscores the importance of considering metabolic disorders in atraumatic tendon ruptures and highlights the diagnostic value of intraoperative findings.

## Introduction

Rupture of the Achilles tendon, although relatively uncommon, is the most frequently ruptured tendon in the lower limb, accounting for approximately 20% of all large tendon injuries.^1^ Individuals with chronic diseases such as systemic lupus erythematosus and rheumatoid arthritis, especially those undergoing long-term corticosteroid therapy, are at increased risk. Spontaneous rupture of tendoachilles has been described in long-term corticosteroid use, fluoroquinolones, or inborn errors of metabolism.^2,3^ In addition, healthy individuals and athletes engaged in physical activities are more susceptible to Achilles tendon ruptures.^4^

Alkaptonuria is a rare metabolic disorder caused by mutations in the HGO gene, leading to a deficiency in homogentisate 1,2-dioxygenase, with an incidence of 1 in 250,000 to 1 in 1,000,000.^3,5^ This enzyme deficiency results in homogentisic acid (HGA) accumulation, which in excess is oxidized to benzoquinones. Polymerized benzoquinones deposit as a dark pigment in various body tissues, a condition known as ochronosis. Usually asymptomatic till adulthood, the most common initial presentation of this disease is ochronotic arthropathy.^5,6^ This pigment deposition leads to the degeneration and destruction of connective tissues,^7^ fragility of bones, early cartilage degeneration, and spontaneous rupture of tendons.^8^ While pigmentation of tendons in both the upper and lower limbs has been reported, there are few documented cases of localized ochronotic pigment deposition in the Achilles tendon resulting in rupture^9^ and nontraumatic calcaneal avulsion in the setting of ochronosis.^8^ Cases with the involvement of the Achilles tendon, quadriceps tendon, and patellar tendon have been reported in the literature.^3^ This paper presents a unique case of bilateral spontaneous calcaneal avulsion in a patient with asymptomatic alkaptonuria.

## Case report

A healthy woman in her late 30s presented with a sudden onset of sharp, shooting pain in the left ankle while walking, accompanied by difficulty bearing weight on the affected limb. General examination was unremarkable. However, a local examination of the left ankle revealed swelling around the Achilles tendon region, severe tenderness, and a palpable gap of 3 cm above the calcaneal insertion. Plantar flexion of the ankle was not possible, and Thompson’s test^10^ was positive. Magnetic resonance imaging (MRI) of the left ankle showed a full-thickness complete tear of the Achilles tendon at its caudal insertional site with a 2.4 cm gap (Kuwada classification^11^—Type II). Achilles tendon repair was performed using a posterior midline approach with a screw anchor. The patient underwent successful rehabilitation and regained the ability to walk without support within 3 months.

**Figure 1.**
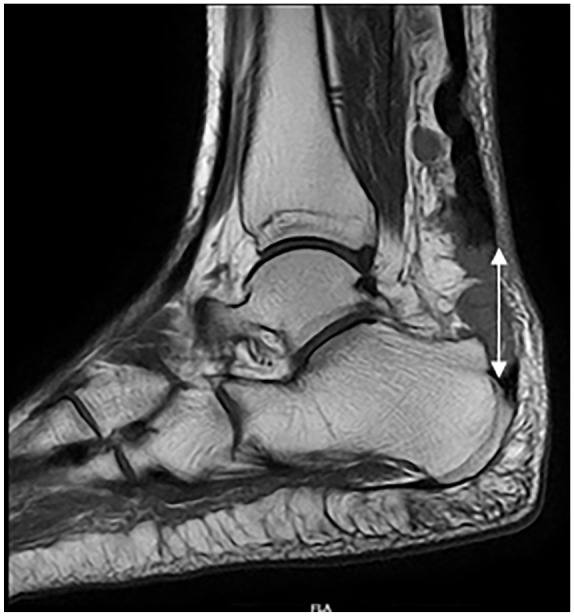
MRI right ankle shows full thickness tear of the tendoachilles with a gap of 3.4 cm.

**Figure 2.**
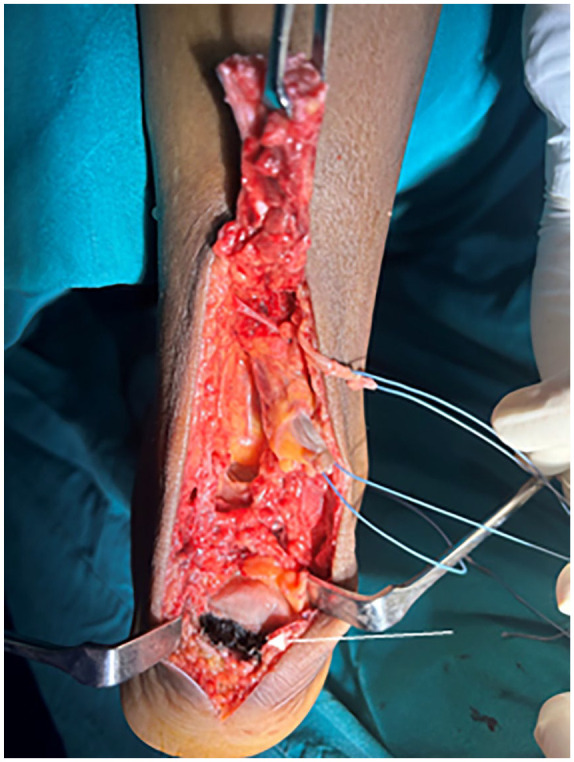
Black pigmentation at the tendoachilles insertion site on the calcaneum.

**Figure 3.**
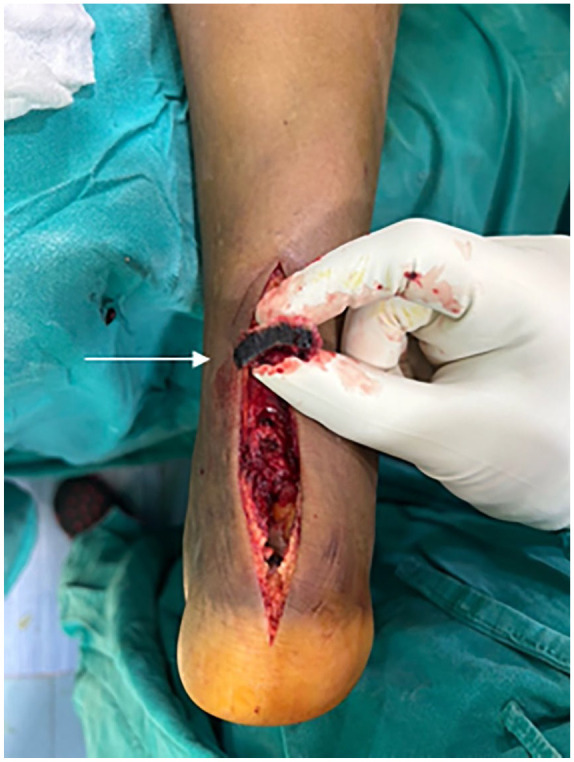
Frayed end of tendoachilles showing black pigment.

**Figure 4.**
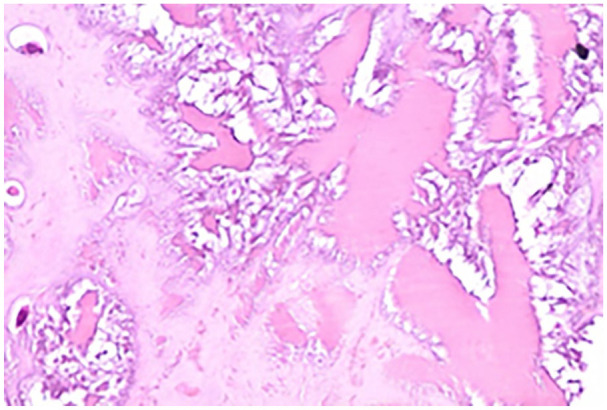
Microscopy of the tendon showing degenerative changes.

Six months later, she developed similar pain in the right ankle while getting onto the bed. This episode was characterized by sharp pain, a pop sound, weakness, and inability to bear weight. Examination revealed findings similar to the previous incident, with a palpable gap of 4 cm just above the insertion of the Achilles tendon. MRI of the right ankle (Figure 1) showed a full-thickness complete tear of the Achilles tendon with a 3.4 cm gap (Kuwada classification^11^—Type III), a partial tear of the anterior talofibular ligament, and tibialis posterior tendinopathy. Using a posterior midline approach, the tendon was exposed. Intraoperatively, the tendon was found to be avulsed from the calcaneal insertion (Figure 2) with the insertion site and cut end of the tendon showing diffuse blackish discoloration (Figure 3). The tissue was sent for histopathological analysis (Figure 4). Due to the inadequate length, the tendon was augmented using the flexor hallucis longus tendon and fixed with a screw anchor. The limb was immobilized using a below-knee plaster of Paris slab in the equinus position.

Sutures were removed on post-op day 14 and the wound displayed superficial dehiscence. Eventually, the wound healed by secondary intention. She is currently undergoing rehabilitation and can mobilize partial weight bearing.

Postoperatively, the HGA result from the urine sample returned positive, confirming the diagnosis of alkaptonuria. NX GEN sequencing for alkaptonuria showed a compound heterozygous mutation—indicating a pathological variant. However, there was no history of blackish discoloration of urine, skin, sclera, or other common presentations of alkaptonuria. The intraoperative discoloration uncovered the underlying alkaptonuria, which facilitated lifestyle modifications and improved the prognosis for the patient.

## Discussion

The Achilles tendon, the strongest tendon in the human body, is frequently ruptured during sports activities that involve sudden, forceful contractions. These ruptures often occur without preceding symptoms and are more commonly observed in individuals with a mean age of 38 years.^12^ In the presented case, the likely mechanism of injury was the sudden dorsiflexion of a plantar-flexed foot. This action, involving the plantar-flexed foot gaining momentum against the ground to push off, results in a forceful contraction of the gastrocsoleus muscle group. The combination of eccentric loads from the contracted gastrocsoleus muscles and the ground reaction force can initiate the rupture of a tendon already weakened by degeneration.

Habusta^1^ noted that spontaneous ruptures are common in degenerated tendons. One significant contributing factor to the degeneration of the Achilles tendon in the patient is the weakening of its collagen structure due to the deposition of ochronotic pigment, as seen in alkaptonuria. Alkaptonuria, an inborn metabolic disorder, leads to the accumulation of HGA, which deposits as a pigment in connective tissues, causing their degeneration and weakening.^5,13^

In this case, the diagnosis of alkaptonuria was suspected intraoperatively based on the unusual black discoloration of the tendon and calcaneal insertion site. This case highlights the importance of considering metabolic disorders in patients presenting with spontaneous tendon ruptures and avulsions, even in the absence of other systemic symptoms. The histopathological analysis confirmed degenerative changes in the Achilles tendon, consistent with ochronosis due to alkaptonuria.

This case is unique due to the intraoperative diagnosis of alkaptonuria in an otherwise asymptomatic woman. The findings underscore the need for a thorough intraoperative examination and histopathological analysis when dealing with atypical presentations of tendon ruptures. Early diagnosis of alkaptonuria can lead to appropriate lifestyle modifications and interventions that can improve patient prognosis and quality of life.

## Conclusion

Alkaptonuria, a metabolic disease, usually asymptomatic till adulthood, presents with a myriad of conditions including, but not limited to arthropathy, tendinopathy, tendon ruptures, pigment deposition in tissues—ochronosis, discoloration of urine, renal stones, and cardiac valve involvement. This case highlights the importance of a high level of clinical suspicion and thorough intraoperative examination and histopathological analysis when dealing with atypical presentations of tendon ruptures. Surgeons must look out for wound complications due to poor wound healing seen in this condition.
